# Predictors of Mortality and Differences in Clinical Features among Patients with Cryptococcosis According to Immune Status

**DOI:** 10.1371/journal.pone.0060431

**Published:** 2013-03-26

**Authors:** Kyle D. Brizendine, John W. Baddley, Peter G. Pappas

**Affiliations:** 1 Department of Infectious Disease, Cleveland Clinic, Cleveland, Ohio, United States of America; 2 Birmingham Veterans Affairs Medical Center, Birmingham, Alabama, United States of America; 3 Division of Infectious Diseases, Department of Medicine, University of Alabama at Birmingham, Birmingham, Alabama, United States of America; Albert Einstein College of Medicine, United States Of America

## Abstract

**Introduction:**

Cryptococcosis is an invasive fungal infection causing substantial morbidity and mortality. Prognostic factors are largely derived from trials conducted prior to the modern era of antifungal and potent combination antiretroviral therapies, immunosuppression, and transplantation. Data describing the clinical features and predictors of mortality in a modern cohort are needed.

**Methods:**

We conducted a retrospective cohort study of patients at our institution diagnosed with cryptococcosis from 1996 through 2010. Data included demographics, clinical features, diagnostics, treatment, and outcomes.

**Results:**

We identified 302 individuals: 108 (36%) human immunodeficiency virus (HIV)-positive, 84 (28%) organ transplant recipients (OTRs), and 110 (36%) non-HIV, non-transplant (NHNT) patients including 39 with no identifiable immunodeficiency. Mean age was 49 years, 203 (67%) were male and 170 (56%) were white. All-cause mortality at 90 days was 21%. In multivariable logistic regression analyses, cryptococcemia (OR 5.09, 95% CI 2.54–10.22) and baseline opening pressure >25 cmH_2_O (OR 2.93, 95% CI 1.25–6.88) were associated with increased odds of mortality; HIV-positive patients (OR 0.46, 95% CI 0.19–1.16) and OTRs (OR 0.46, 95% CI 0.21–1.05) had lower odds of death compared to NHNT patients.

**Conclusions:**

Predictors of mortality from cryptococcosis in the modern period include cryptococcemia, high intracranial pressure, and NHNT status while drug(s) used for induction and historical prognostic factors including organ failure syndromes and hematologic malignancy were not associated with mortality.

## Introduction

Cryptococcosis is an important opportunistic fungal infection causing considerable morbidity and mortality among immunocompromised patients including individuals positive for human immunodeficiency virus (HIV), organ transplant recipients (OTRs), and other immunocompromised patients such as those with sarcoidosis, immunoglobulin disorders, chronic glucocorticoid treatment, disorders characterized by dysfunction of cell-mediated immunity, conditions requiring the use of anti-tumor necrosis factor-α therapy and other disease-modifying agents, and hematologic malignancy [1]–[5]. Occasionally, otherwise normal individuals develop serious consequences of invasive disease. It is estimated that approximately 1 million cases and 625,000 deaths occur per year worldwide due to central nervous system (CNS) cryptococcosis among HIV-infected individuals [6]. In comparison, there are few estimates of the burden of cryptococcal disease among non-HIV-infected patients.

Prognostic factors, which inform treatment recommendations in the clinical practice guidelines for management [7], are mostly derived from results of trials conducted in an earlier era of treatment and risk [8]–[11]. Since that time, medical and surgical advancements have ushered in a period with a rapidly growing at-risk population of patients who frequently receive specific immunosuppression. This new period includes the introduction of triazole antifungal therapy, widespread use of potent combination antiretroviral therapy (cART), institution of steroid-sparing immunosuppression including calcineurin inhibitors and biologic agents, and expansion of both solid organ (SOT) and hematopoietic stem cell transplantation (HSCT). For example, the clinical features and outcomes of cryptococcosis that develop following induction therapy with alemtuzumab and tacrolimus for prevention of acute organ rejection may differ compared to patients from an earlier period managed with a glucocorticoid-based regimen. Few series have been published recently comparing groups of patients according to immune status, but they are limited in scope or number of patients [12]–[15]. More robust data on the epidemiology and prognostic factors of cryptococcosis in the current era are needed.

Herein we review cases of cryptococcosis occurring at our institution from 1996–2010 with two specific aims: 1) to describe the epidemiology of cryptococcosis at our center and 2) to characterize prognostic factors associated with death.

## Methods

### Ethics statement

As the institution had routinely collected the data for patient care and as analysis of the data posed minimal risk, this protocol was approved by the UAB Institutional Review Board for human subjects with a waiver of informed consent.

We identified cases of cryptococcosis diagnosed antemortem at the University of Alabama at Birmingham (UAB) from 1 January 1996 through 31 December 2010 by review of microbiology and serology reports, International Classification of Diseases, 9th Revision (ICD-9) codes, and outpatient Infectious Diseases clinic records. The study start date was chosen to minimize confounding in the associations with mortality due to untreated HIV infection or other opportunistic infections by limiting the cohort to those HIV-positive patients with the opportunity to receive cART. A standardized case report form was used to collect data on age, sex, race, site of involvement, underlying disease, clinical presentation, length of symptoms, diagnostic laboratory and radiographic results, medical treatment, interventions, and outcomes.

### Definitions

A case was defined by a positive culture for *Cryptococcus* spp. from blood, body fluid, tissue, or sputum, or a positive serum or cerebrospinal fluid (CSF) cryptococcal antigen (CrAg) assay with a compatible clinical and/or radiographic presentation. Sites of involvement were classified as follows: 1) central nervous system (CNS), which included meningeal and parenchymal brain involvement, included patients with infection of the CNS alone and encompassed those with concomitant CNS and pulmonary disease; 2) pulmonary, which included disease limited to the lungs, pleura, and/or pleural fluid but not CNS; 3) blood, which involved any isolation of *Cryptococcus* spp. in blood culture; and 4) other. To determine that cryptococcosis was the etiology of pulmonary disease, patients had to demonstrate a positive serum CrAg or culture of blood, pleural fluid, lung tissue, or sputum with a compatible abnormal chest radiograph with nodules, lobar consolidation, interstitial pattern, or ARDS. In the presence of concomitant CNS disease, patients had to demonstrate a compatible abnormal chest radiograph, but no additional pulmonary sampling was required. A single patient may have more than one site of involvement. For example, a patient with pulmonary nodules and a positive blood culture and CSF CrAg was classified as blood and CNS sites of involvement whereas a patient with pulmonary nodules and a positive blood culture was classified as pulmonary and blood sites. Time to diagnosis was defined by the length of symptoms measured in days. It corresponded to the number of days between symptom onset and meeting the case definition.

Patients were categorized into the following 3 groups based upon immune status: HIV-positive, OTRs, and NHNT patients. Predisposing conditions were defined as follows: *chronic steroid use* was the daily use of more than 5 mg prednisone equivalent for >30 days preceding the date of diagnosis of cryptococcosis; *renal insufficiency/end-stage renal disease* (ESRD) was either a glomerular filtration rate (GFR) 15–60 mL/min/1.73 m^2^ that persisted for more than 90 days (renal insufficiency) or GFR <15 mL/min/1.73 m^2^ or dialysis within 90 days (ESRD) preceding the date of diagnosis of cryptococcosis; *cancer* included hematologic malignancies and malignant solid tumors documented prior to the diagnosis of cryptococcosis; and *rheumatologic disease* was the presence of rheumatoid arthritis, lupus, psoriatic arthritis, ankylosing spondylitis, Sjogren's syndrome, or inflammatory myopathy. Patients with no identifiable predisposing conditions who had negative testing for CD4+ lymphocytopenia and immunoglobulin deficiency were classified as having no underlying disease.

Antifungal therapy was classified according to drug(s) administered for induction, consolidation, and chronic suppression. Induction was defined by the initial drug(s) given for ≥3 days. Consolidation was defined as the therapy given following a response to the induction regimen. Chronic suppression represented the ongoing maintenance antifungal therapy given to patients without evidence of active disease. The duration of antifungal therapy and the average daily dose were recorded.

The primary outcome was 90-day all-cause mortality. Attributable mortality due to cryptococcosis was also determined. It included patients who died without a response to therapy or died of any acute event involving their site of infection. Follow up began on the date of diagnosis and ended on the date of death or the date of the last available record.

### Statistical analysis

Descriptive statistics were computed, and differences in characteristics across groups were analyzed by chi-square test or analysis of variance techniques. Multivariable analyses for prognostic factors associated with 90-day mortality were performed using stepwise multiple logistic regression analysis. The criterion for entry into the model was significance at the α = 0.20 level while the criterion for remaining in the model was significance at the α = 0.05 level. Odds ratios and corresponding 95% confidence intervals were calculated. Model fit was assessed using the Hosmer-Lemeshow goodness-of-fit statistic, and the model fit the data well. All statistical tests were two-tailed and utilized a 5% significance level. Analyses were performed using SAS software version 9.2 (SAS Institute Inc, Cary, North Carolina).

## Results

### Patient characteristics

**Figure 1:**
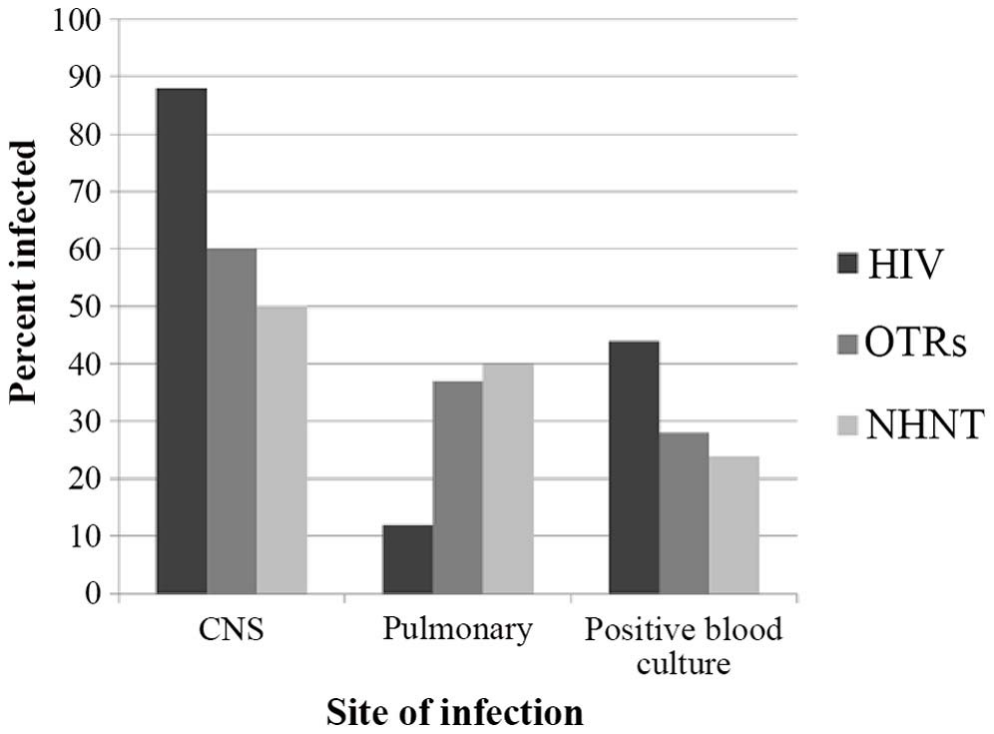
Differential site of infection by host immune status among 302 patients with cryptococcosis at UAB, 1996–2010. HIV-positive patients presented with significantly more CNS disease and cryptococcemia compared to OTRs and NHNT patients, who more often demonstrated disease confined to the lungs. Note. HIV, human immunodeficiency virus; OTRs, organ transplant recipients; NHNT, non-HIV, non-transplant; CNS, central nervous system.

During the study period, there were 302 patients diagnosed with cryptococcosis at UAB. One hundred eight (36%) were HIV-positive, 84 (28%) were OTRs, and 110 (36%) were NHNT patients. Among approximately 200 viable cultures available for subsequent testing, there were 2 isolates of *C. gattii*. The remaining isolates were *C. neoformans*. As illustrated in Table 1, the mean time to diagnosis among NHNT patients was 68 days, significantly longer compared to HIV-positive (22) and OTRs (26) (p<0.001). HIV-positive patients were significantly younger and composed of a greater proportion of African-American men (all p<0.05), features consistent with the epidemiology of patients in care for HIV at our institution. They were more likely to present with headache, altered mental status, and visual changes (all p<0.05) in accordance with their greater proportion of CNS disease and cryptococcemia compared to OTRs and NHNT patients, who, as shown in Figure 1, more often demonstrated disease confined to the lungs (all p<0.05). The distribution of OTRs delineated in Table 1 reflects the activity of the transplant programs at our institution. A higher proportion of an organ type does not equate to risk of infection. Among patients with CNS disease, 38/95 (40%) HIV-positive patients demonstrated baseline opening pressures >25 cmH_2_O compared with 11/50 (22%) OTRs and 13/55 (24%) NHNT patients (p<0.001).

**Table 1:** Characteristics of 302 patients with cryptococcosis at UAB, 1996–2010.

	HIVN = 108 (%)	OTRN = 84 (%)	NHNTN = 110 (%)	*P*–value	Total cohort N = 302 (%)
Mean age (± SD), years	39 (9.8)	54 (11.9)	56 (15.3)	<0.001	49 (12.6)
Male gender (%)	84 (78)	52 (62)	67 (61)	0.012	203 (67)
White (%)	23 (21)	66 (79)	83 (75)	<0.001	172 (57)
African American (%)	83 (77)	18 (22)	22 (20)	<0.001	123 (41)
Mean time to diagnosis, days	22	26	68	<0.001	40
Transplant type (%)					
Kidney	0 (0)	46 (55)	n/a	n/a	46 (15)
Liver	0 (0)	17 (20)	n/a	n/a	17 (6)
Heart	0 (0)	15 (18)	n/a	n/a	15 (5)
Lung	0 (0)	9 (11)	n/a	n/a	9 (3)
Pancreas	0 (0)	5 (6)	n/a	n/a	5 (2)
HSCT	0 (0)	2 (2)	n/a	n/a	2 (0.7)
Underlying disease (%)					
None	n/a	n/a	39 (36)	n/a	39 (13)
Steroids	0 (0)	73 (88)	27 (25)	<0.001	100 (33)
Renal insufficiency/ESRD	3 (3)	36 (43)	7 (6)	<0.001	46 (15)
Cancer	1 (1)	5 (6)	31 (28)	<0.001	37 (12)
Diabetes mellitus	4 (4)	21 (25)	13 (12)	<0.001	38 (13)
Rheumatologic disease	0 (0)	1 (1)	7 (6)	0.008	8 (3)
Cirrhosis	0 (0)	11 (13)	5 (5)	<0.001	16 (5)
Site of infection (%)					
Central nervous system	95 (88)	50 (60)	55 (50)	<0.001	200 (66)
Bloodstream	47 (44)	23 (28)	26 (24)	0.005	96 (32)
Pulmonary	13 (12)	31 (37)	44 (40)	<0.001	88 (29)
Cutaneous	4 (4)	8 (10)	2 (2)	0.033	14 (5)
Bone and joint	0 (0)	1 (1)	2 (2)	0.388	3 (1)
Soft tissue	0 (0)	2 (2)	1 (1)	0.251	3 (1)
Clinical presentation (%)					
Fever	44 (41)	33 (40)	31 (28)	0.119	108 (36)
Malaise	23 (21)	25 (30)	26 (24)	0.363	74 (25)
Weight loss	26 (24)	11 (13)	19 (17)	0.150	56 (19)
Headache	71 (66)	36 (43)	44 (40)	<0.001	151 (50)
Altered mental status	43 (40)	25 (30)	27 (25)	0.039	95 (31)
Visual changes	24 (22)	5 (6)	13 (12)	0.004	42 (14)
Cranial nerve palsy	9 (8)	3 (4)	9 (8)	0.364	21 (7)
Cough	15 (14)	20 (24)	25 (23)	0.137	60 (20)
Dyspnea	9 (8)	16 (19)	30 (28)	0.001	55 (18)
Diagnostics (%)					
Serum CRAG ≥ 1:512	40 (37)	24 (29)	16 (15)	0.001	80 (27)
Mean CSF WBC count, /mm3	65	91	201	0.026	106
CSF CRAG ≥ 1:512	40 (37)	17 (20)	20 (18)	0.003	77 (26)
CSF OP > 25 cm H_2_O	38 (35)	11 (13)	13 (12)	<0.001	62 (21)
Mortality (%)					
Ninety-day mortality	20 (19)	14 (17)	29 (27)	0.190	63 (21)
One-year mortality	28 (26)	20 (24)	38 (35)	0.193	86 (28)

NOTE. HIV, human immunodeficiency virus; OTR, organ transplant recipient; NHNT, non-HIV, non-transplant; HSCT, hematopoietic stem cell transplant; ESRD, end-stage renal disease; CRAG, cryptococcal antigen; CSF, cerebrospinal fluid; OP, opening pressure

### Laboratory and radiographic findings

Median serum CrAg was 1∶256 (IQR 1:16–1:2048) and median CSF CrAg 1∶256 (IQR 1∶16–1∶2048). Among those with CNS disease, mean CSF cell count among NHNT patients was 201 per mm^3^, significantly higher compared to HIV-positive patients (65) and OTRs (91) (p = 0.026), while mean CSF glucose was 39 mg/dL, significantly lower than HIV-positive patients (46) and OTRs (53) (p = 0.038). NHNT patients demonstrated the highest mean CSF protein, 185 mg/dL, but it was not significantly different compared to HIV-positive patients (133) and OTRs (108) (p = 0.297). The proportion of patients with positive CSF cultures was similar across groups, ranging from 82 to 86% (p = 0.722). Among 169 patients with baseline head computed tomography (CT), 151 (89%) had CNS cryptococcosis. Twenty-one (13%) patients demonstrated hydrocephalus, 4 (2%) mass lesions, and 144 (85%) were normal. Two hundred eighty-two (93%) patients had baseline plain chest radiographs of which 171 (59%) were normal. Abnormalities included 34 (12%) patients with lobar consolidation, 30 (11%) with a diffuse interstitial pattern, 26 (9%) unilateral nodules, 14 (5%) bilateral nodules, 18 (6%) pleural effusions, and 4 (1%) with cavitation. Ninety-six patients underwent chest CT demonstrating pulmonary nodules in 8/16 (50%) HIV-positive patients, 22/32 (69%) OTRs, and 29/48 (60%) NHNT patients. Cavitary lesions were noted in 3/16 (19%) HIV-positive patients, 4/32 (13%) OTRs, and 8/48 (17%) NHNT patients.

### Treatment and outcome

For induction therapy, 149 (49%) patients received a combination of either amphotericin B deoxycholate (AmBd) or a lipid formulation of amphotericin B (LFAmB) plus flucytosine (5-FC); of these, 87 (58%) received combination therapy with AmBd plus 5-FC and 62 (42%) with LFAmB plus 5-FC. Nineteen (6%) patients received a combination of AmBd or LFAmB plus fluconazole. Finally, 71 (24%) received fluconazole alone, among whom 54 (76%) had non-CNS disease. The remainder of the patients (30 [10%] with CNS disease and 33 [11%] with non-CNS disease) received induction with an AmB product alone or no induction. There was little variability in drugs used for consolidation and chronic suppression: most patients received 200–400 mg average daily dose of fluconazole. Comparative effectiveness of induction regimens with combination therapy, as measured by 90-day mortality, related to site of infection is presented in Table 2. For patients with CNS disease, there was no significant difference in outcomes. For patients with non-CNS disease, those receiving fluconazole alone or in combination with either AmBd or LFAmB for induction had the lowest mortality.

**Table 2:** Comparative effectiveness of induction with combination therapy, as measured by 90-day mortality, related to site of infection among 302 patients with cryptococcosis at UAB, 1996–2010.

Site of infection	AmBd or LFAmB + 5-FC N = 149 (%)	AmBd + 5-FCN = 87 (%)	LFAmB + 5-FCN = 62 (%)	AmBd or LFAmB + Flu N = 19 (%)	FluN = 71 (%)
Non-CNS, N = 102	13 (13)	7 (7)	6 (6)	2 (2)	54 (53)
90-day mortality	5 (38)	1 (14)	4 (67)	0 (0)	6 (11)
CNS, N = 200	136 (68)	80 (40)	56 (28)	17 (9)	17 (9)
90-day mortality	30 (22)	16 (20)	14 (25)	3 (18)	4 (24)

NOTE. Non-CNS site of infection denotes any site without CNS involvement. CNS site of infection includes patients with CNS only and CNS and non-CNS infection concomitantly. AmBd, amphotericin B deoxycholate; LFAmB, lipid formulation amphotericin B; 5-FC, flucytosine; Flu, fluconazole; CNS, central nervous system

### Prognostic factors

**Figure 2:**
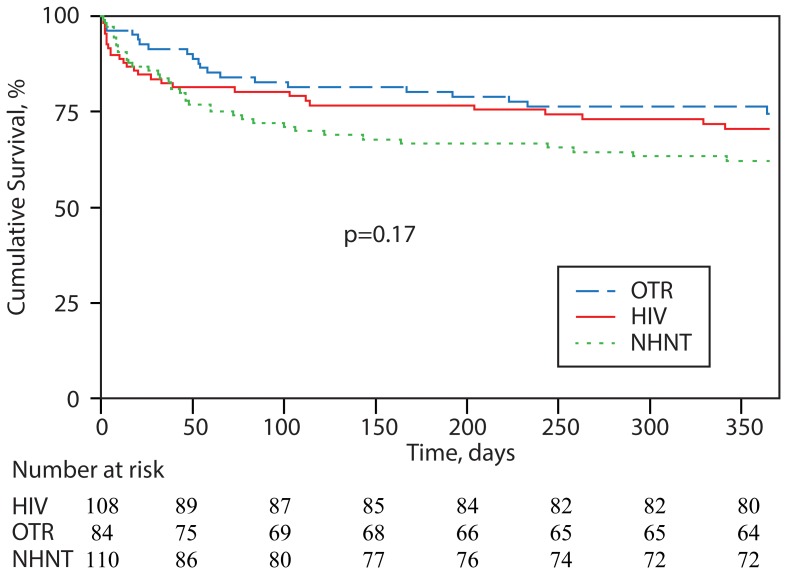
Kaplan-Meier survival curve of HIV-positive patients, OTRs, and NHNT patients among 302 patients with cryptococcosis at UAB, 1996–2010. Non-HIV, non-transplant patients had the lowest survival, but the difference across groups did not reach statistical significance. Note. HIV, human immunodeficiency virus; OTRs, organ transplant recipients; NHNT, non-HIV, non-transplant.

Among all patients, 90-day mortality was 21%. Among those deaths with available data (n = 55), 42 (76%) were attributable to cryptococcosis. At one year of follow up, among 71 deaths with available data, 48 (68%) were attributable to cryptococcosis. Mortality was highest in the NHNT group (27%), but this did not reach statistical significance across groups (p = 0.190). The Kaplan-Meier survival curves are shown in Figure 2. On univariate analyses, prognostic factors positively associated with 90-day mortality included cancer (p = 0.018), fever (p = 0.031), altered mental status (p = 0.001), positive blood cultures (p<0·001), and high (≥1:512) serum CrAg (p = 0.021). Demographic and clinical features negatively associated with 90-day mortality were age <50 (p = 0.020), presenting with headache (p = 0.003) or cough (p = 0.047), and pulmonary site of infection (p = 0.027).

In multivariable logistic regression analyses, cryptococcemia (OR 5.09, 95% CI 2.54–10.22; p<0.001)and baseline opening pressure >25 cmH_2_O (OR 2.93, 95% CI 1.25–6.88; p = 0.013) were associated with increased odds of mortality (Table 3). Presenting with altered mental status (OR 1.96, 95% CI 0.98–3.91; p = 0.057) was associated with higher odds of death, but the association did not reach statistical significance. In contrast, age <50 (OR 0.42, 95% CI 0.20–0.92; p = 0.029) and headache (OR 0.33, 95% CI 0.16–0.68; p = 0.003) were protective. Choice of induction therapy, organ transplant type, and organ failure syndromes were not significant prognostic factors and were not retained in any of the multiple models evaluated (data not shown). Finally, too few patients received any one specific biologic immunosuppressive agent to permit meaningful comparisons among drugs in that class. Therefore, no individual agent could be shown to be associated with mortality in any of the multiple models evaluated (data not shown).

**Table 3:** Results of multivariable analyses of predictors of 90-day mortality among 302 patients with cryptococcosis at UAB, 1996–2010.

Variable	OR	95% CI	*P*–value
Cryptococcemia	5.09	2.54 – 10.22	<0.001
Baseline CSF opening pressure > 25 cm H_2_O	2.93	1.25 – 6.88	0.013
Pulmonary cryptococcosis	0.44	0.18 – 1.07	0.070
Age < 50	0.42	0.20 – 0.92	0.029
Headache	0.33	0.16 – 0.68	0.003
Altered mental status	1.96	0.98 – 3.91	0.057
HIV versus NHNT	0.46	0.19 – 1.16	0.111
OTRs versus NHNT	0.46	0.21 – 1.05	0.111

NOTE. CSF, cerebrospinal fluid; HIV, human immunodeficiency virus; NHNT, non-HIV, non-transplant; OTRs, organ transplant recipients

## Discussion

Our study is the largest single-site series of well-described, contemporary cases of cryptococcosis in a heterogeneous population to address the prognostic factors associated with mortality. We observed that patients with cryptococcemia, high intracranial pressure, and altered mental status had increased odds of dying. Patients who were under the age of 50 and those who presented with headache had reduced odds of death. HIV-positive patients and OTRs demonstrated decreased odds of mortality compared to NHNT patients. We further documented significant differences in clinical features across groups categorized by immune status. HIV-positive patients exhibited more CNS disease and positive blood cultures than OTRs and NHNT patients. They were also more likely to have elevated baseline opening pressure and high CSF CrAg. NHNT patients exhibited greater CSF pleocytosis, protein concentration, and hypoglycorrhachia, representing a more robust inflammatory response compared to the other groups. Finally, we identified 39 patients with no identifiable immunodeficiency with cryptococcosis whose characterization and comparison to immunocompromised patients are intriguing and require much more detailed analysis.

Prior to the global HIV pandemic, cryptococcosis was recognized to be associated with dysfunction of cell-mediated immunity, and subsequently, HIV-associated disease has been well documented. The emerging prominent group of NHNT patients has been far less studied. One substantial contribution of our work involves the detailed description and analysis of that important group in the modern era. The time to diagnosis among NHNT patients was extremely prolonged, and it has been shown that delays in diagnosis and treatment were associated with poor outcomes [16]. Indeed, our results showed NHNT patients demonstrated the highest mortality at both 90 days and one year. This extended time to diagnosis possibly accounts for the higher mortality at 90 days, but we suspect the differences in one-year mortality are due to the NHNT patients’ other underlying diseases. Considering the 71 immunocompromised NHNT patients and excluding the 39 individuals with no identifiable immunodeficiency, we found that 90-day mortality was 35% (25/71), which approximated research settings in sub-Saharan Africa (24–37%) [17], [18]. These findings were also consistent with recent retrospective data from a smaller U.S. cohort that found 37% mortality among NHNT patients whose mean duration of symptoms was longer than the comparator groups of HIV-positive patients and OTRs [19]. Work from Taiwan also showed higher mortality (30 vs. 5%, p = 0.029) and longer symptom duration among HIV-negative patients with meningitis compared to HIV-positive patients [20]. That protracted time to diagnosis may lead to additional morbidity. Stroke, blindness, deafness, neurologic disability, and cognitive dysfunction, for example, may disproportionately affect NHNT patients due in part to delayed diagnosis and treatment, a possibility that needs to be explored.

Our results examining other differences across patient groups based upon immune status were generally in agreement with recent studies with an important exception for the proportion of patients with cryptococcemia. It has been shown by utilizing the Prospective Antifungal Therapy Alliance (PATH) registry in North America that 74/88 (84%) HIV-positive patients studied had CNS disease [13]. Nearly the same proportion has been found in a recent analysis of a cohort from the Southeast U.S., which demonstrated that 74/86 (86%) HIV-positive patients examined had CNS disease [19]. In studies from Thailand and Taiwan, it has been shown that 137/149 (92%) and 10/10 (100%) HIV-positive patients evaluated had CNS disease, respectively [14], [21]. Pulmonary involvement was more common among OTRs and NHNT patients in those studies, but there was no difference in the proportion of positive blood cultures between HIV-positive and HIV-negative patients. In contrast, our data showed significantly more cryptococcemia among HIV-positive patients, which is consistent with a comparison of 19 HIV-positive patients to 53 HIV-negative patients in Taiwan that found significantly more cryptococcemia in HIV-positive patients (53 vs. 21%, p = 0.009) [20]. This finding was not trivial given that the strongest association with 90-day mortality was a positive blood culture. The size of our cohort and robust clinical microbiology and mycology laboratory support at our institution may explain how differences in the proportion of patients with cryptococcemia emerged. With regard to other diagnostic findings including higher CSF CrAg titer and elevated opening pressure among HIV-infected patients compared to OTRs and NHNT patients, our results were consistent with published studies [14], [15], [19]–[21]. Taken together, the above observations underscore the importance of considering host factors in cryptococcosis as a critical step in understanding prognostic factors associated with mortality.

In our data, the association of cryptococcemia with poorer outcome is perhaps not surprising given its correlation with more severe and/or disseminated disease [22]–[25]. The association between high intracranial pressure, the aggressive management of which is known to be crucial, and mortality is expected; however, presenting with headache, a feature associated with elevated pressure, was associated with lower odds of mortality. It was possible patients sufficiently cognitively intact to complain of headache suffered less severe disease than those who presented with altered mental status incapable of such a complaint. The results of our multivariable model were in agreement with prior studies that showed altered mental status increased the odds of dying while younger age was protective [2], [26]–[28].

Perhaps the most remarkable finding from our data involves the clinical features and treatment factors that were not associated with mortality. In studies from an earlier era, it has been shown that organ failure syndromes, such as renal insufficiency and cirrhosis, and hematologic malignancy predicted mortality among 306 HIV-negative patients from 15 U.S. medical centers diagnosed with cryptococcosis from 1990–1996 [2]. It has additionally been shown in an analysis of 52 patients in Taiwan with cryptococcemia diagnosed from 1981–2001 that cirrhosis and severity of sepsis were associated with 30-day mortality [23]. A literature review of cryptococcosis in OTRs published through 1998 found that only renal failure on admission was predictive of death among 172 patients [24]. Work in France evaluating 230 patients enrolled from 1997–2001 identified neurological signs and symptoms, abnormal neuroimaging, and hematological malignancy as predictors of 90-day mortality [26], [27]. All patients in these series were diagnosed prior to 2002. Neither organ failure syndromes nor hematologic malignancy was retained in the multiple models we tested, suggesting a diminished role in predicting mortality and emphasizing differences between an older and more contemporary period. It is possible that improved medical and surgical care for these conditions including renal replacement therapy, expansion of liver transplant programs and refinements in surgical technique, utilization of nonmyeloablative chemotherapy, and use of oral triazole antifungal prophylaxis all combined to diminish the influence of these conditions on short-term (90-day) mortality.

Finally, results of our multivariable analyses found no association between drug(s) used for induction and mortality, which is in contrast to the study by Sun and colleagues in which patients who received LFAmB had reduced odds of dying [29]. In that study restricted to OTRs, 37/55 (67%) LFAmB recipients received combination therapy with 5-FC, a significantly higher proportion compared to 8/20 (40%) AmBd recipients (p = 0.033). Studies in varied patient populations have shown a beneficial role for combination therapy with 5-FC [8], [11], [24], [30], [31], whose differential administration in that study may partially account for the apparent benefit of LFAmB. Our data comparing 136 patients with CNS disease who received a combination of AmBd plus 5-FC or LFAmB plus 5-FC revealed similar mortality (20 vs. 25%; p = 0.454) (Table 2).

The analysis of our large, heterogeneous modern cohort provides advantages over prior studies, but the observational design was limited principally by confounding by indication, and results of some comparisons should be interpreted cautiously. For example, patients with non-CNS disease who received fluconazole alone had the lowest mortality, almost certainly reflecting less severe disease rather than therapeutic superiority [7]. Clinicians’ perception of patients’ severity of illness may have led to systematically different management, which could neither be controlled for nor captured. Although we measured a number of covariates, unmeasured variables remained. As a tertiary referral center within the Southeast U.S., our medical center manages patients with more complicated and co-morbid conditions, representing a referral bias. These data also reflected the biases of the clinicians at our institution, but limiting the cohort initially to a single center increased internal validity. Another important advantage of our study involved the employment of 90-day mortality as the primary outcome rather than a surrogate microbiologic endpoint, which may not directly translate into patient-perceived benefit and harm.

Although significant attention has been justifiably focused on the burden of HIV-associated cryptococcosis in sub-Saharan Africa and much of the developing world, the present study reinforces that cryptococcal infections continue to be associated with substantial mortality among a contemporary group of patients in the U.S. Our observations provide valuable insights into the predictors of mortality and identify important differences across groups based upon immune status. Moreover, they may serve in refining management recommendations and for fostering earlier recognition of cryptococcosis. Data analyzing predictors of death and other important outcome measures such as neurologic disability based on host immune status are needed.
